# Fight or flee, a vital choice for *Clostridioides difficile*

**DOI:** 10.1002/mlf2.12102

**Published:** 2024-02-09

**Authors:** Ji Zeng, Shuying Fang, Jinquan Guo, Min Dong, Guo‐Bao Tian, Liang Tao

**Affiliations:** ^1^ School of Biomedical and Pharmaceutical Sciences Guangdong University of Technology Guangzhou China; ^2^ Department of Brest Surgery Panyu Central Hospital Guangzhou China; ^3^ Department of Microbiology Harvard Medical School Boston Massachusetts USA; ^4^ Department of Urology, Boston Children's Hospital Harvard Medical School Boston Massachusetts USA; ^5^ Department of Microbiology Zhongshan School of Medicine, Sun Yat‐sen University Guangzhou China; ^6^ Advanced Medical Technology Center, The First Affiliated Hospital, Zhongshan School of Medicine Sun Yat‐sen University Guangzhou China; ^7^ Key Laboratory of Tropical Diseases Control (Sun Yat‐sen University), Ministry of Education Guangzhou China; ^8^ School of Medicine Xizang Minzu University Xianyang China; ^9^ Center for Infectious Disease Research, Westlake Laboratory of Life Sciences and Biomedicine Westlake University Hangzhou China; ^10^ Research Center for Industries of the Future, School of Life Sciences Westlake University Hangzhou China

**Keywords:** *C. difficile*, sporulation, TcdA, TcdB, toxin production

## Abstract

*Clostridioides difficile* is a leading cause of healthcare‐associated infections, causing billions of economic losses every year. Its symptoms range from mild diarrhea to life‐threatening damage to the colon. Transmission and recurrence of *C. difficile* infection (CDI) are mediated by the metabolically dormant spores, while the virulence of *C. difficile* is mainly due to the two large clostridial toxins, TcdA and TcdB. Producing toxins or forming spores are two different strategies for *C. difficile* to cope with harsh environmental conditions. It is of great significance to understand the molecular mechanisms for *C. difficile* to skew to either of the cellular processes. Here, we summarize the current understanding of the regulation and connections between toxin production and sporulation in *C. difficile* and further discuss the potential solutions for yet‐to‐be‐answered questions.

## INTRODUCTION

*Clostridioides difficile* is an obligate, anaerobic, Gram‐positive bacterium that can produce toxins and form spores^1^. *C. difficile* infection (CDI) has been listed as an urgent threat of antibiotic resistance by the Centers for Disease Control and Prevention (CDC)^2^. About 0.25–0.5 million infection cases are diagnosed in America every year, including 13,000–20,000 in‐hospital deaths^1^, ^2^, ^3^. The first line of treatment for CDI is antibiotics; however, about 15%–35% of patients relapse, and ~40%–65% of relapsed patients would have multiple recurrences^3^, ^4^. The last resort for treating recurrent CDI is fecal microbiota transplantation (FMT). However, the safety of FMT is under debate^5^. Consequently, a better and safer therapy for recurrent CDI is always in demand.

**Figure 1:**
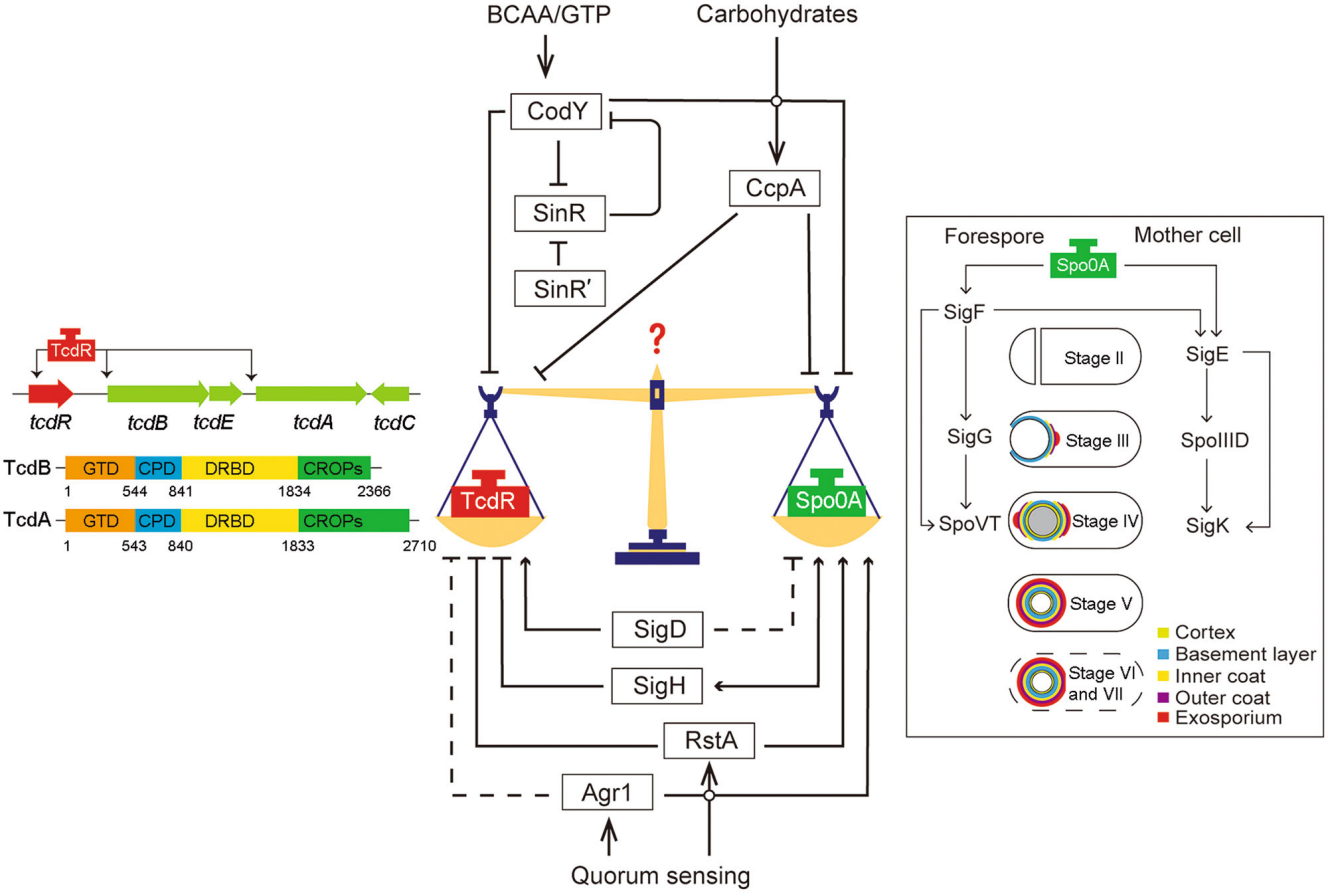
The regulatory network between sporulation and toxin production. The central panel depicts important regulatory factors. CcpA, CodY, and SinR tune sporulation and toxin production codirectionally, while SigD, SigH, RstA, and Agr1 tune these two processes differentially. TcdR and Spo0A possibly suppress each other. Arrows indicate upregulation; the blunt ended line indicates downregulation; the dashed line indicates uncertainty or conflict reports. The left panel depicts the *Clostridioides difficile* PaLoc and the domains of TcdA and TcdB. The right panel depicts the stage II to stage VII of spore formation and the regulators in the forespore and mother cell.

The virulence is mainly contributed by two large clostridial toxins, toxin A (TcdA) and toxin B (TcdB), and, in some strains, the third binary toxin CDT^2^, ^6^, ^7^. TcdA and TcdB contain an N‐terminal glucosyltransferase domain (GTD), a cysteine protease domain (CPD), an intermingled membrane translocation delivery and receptor‐binding domain (DRBD), and a C‐terminal combined repetitive oligopeptides (CROPs) domain (Figure 1)^2^. The two toxins enter the cell via receptor‐mediated endocytosis^8^, ^9^. Translocation of the GTD and CPD domains is then induced by the reduction of pH within endosomes, followed by autoproteolytic cleavage of the GTD domain that glycosylates and inactivates small GTPases^2^. The severity of the illness of CDI is directly associated with the production of toxins. The *tcdA* and *tcdB* genes are located in a 19.6 kb pathogenic locus (PaLoc), which also contains *tcdR*, *tcdE*, and *tcdC* (Figure 1)^10^, ^11^. TcdR is a sigma factor that binds to RNA polymerase to promote the expression of *tcdA* and *tcdB* ^10^, ^11^. TcdC is an anti‐sigma factor of TcdR, while TcdE may be involved in the secretion of toxins^12^. Toxin expression is largely determined by TcdR, which is regulated, directly or indirectly, by CcpA, CodY, and so forth^10^, ^11^. The *C. difficile* transferase (CDT) is a pore‐forming toxin that catalyzes the ADP‐ribosylation of actin^7^. CdtR, a LytTR family regulator within the CDT locus (CDTLoc), positively regulates CDT production^7^. However, little is known about other regulatory factors to CDTLoc^7^.

It is commonly believed that the high recurrence and transmission of *C. difficile* are generally due to the metabolically inactive spores, which may persist in gut epithelial cells and thus serve as a source of recurrence^13^. Spores are highly resistant to multiple factors, such as heat, UV light, and chemical insults^14^. The spore is composed of an inner partially dehydrated core, inner membrane, germ cell wall, cortex peptidoglycan layer, outer membrane, spore coat, and outer exosporium^15^. Sporulation in *C. difficile* has been arbitrarily divided into seven stages (Figure 1)^3^, ^15^. At stage 0, *C. difficile* integrates complex environmental signals and phosphorylates the sporulation master regulator Spo0A. Upon phosphorylation, Spo0A upregulates early‐sporulation genes and starts the irreversible genetic program. At stages I and II, the *C. difficile* genome duplicates, and a polar septum divides the *C. difficile* cell into a larger mother cell and a smaller forespore. From stage III to stage V, the mother cell engulfs the forespore, a thick layer of cortex peptidoglycan is synthesized between the two membranes surrounding the forespore, the cortex layer is modified, and many proteins localize and spread along the forespore outer membrane to build the proteinaceous spore coat. At stages VI and VII, the outermost exosporium layer forms, the mother cell is lysed, and the forespore is finally released into the environment (Figure 1).

Toxin production and sporulation are two actions taken by *C. difficile* when nutrients are limited^2^, ^3^. As both processes consume a large amount of energy and resources, it is critical for *C. difficile* to make the right decision at the right time. A wrong decision affects not only the fate of a single cell but also the integrity and survival rate of the bacterial population^2^, ^16^. Quite a few reports suggest an existing linkage between these two bacterial processes. Yet, it is not clear whether these two processes are mutually exclusive and what the molecular mechanisms are for bacteria. Characterization of the molecular mechanism would help to design the treatment protocol during infection. In the following part, we summarize important regulatory factors that tune toxin production and sporulation similarly or differentially, which may provide new insights into the pathogenesis of *C. difficile*, and treatments of CDI and recurrent infections.

## REGULATORS CONTROL TOXIN PRODUCTION AND SPORULATION CODIRECTIONALLY

### CcpA

CcpA is a LacI/GalR family protein, composed of a signal‐receiving and dimerization domain and a DNA binding domain^17^. It is the master regulator of carbon catabolite repression (CCR), a mechanism to control the sequential consumption of carbohydrates in low G+C Gram‐positive bacteria^17^. The active transport of carbohydrates is achieved by a phosphoenolpyruvate‐dependent phosphotransferase system (PTS), composed of enzyme I (EI), histidine‐containing phospho‐carrier protein (HPr), and enzyme II (EII). In the presence of the preferred carbon source, glucose, for example, the PTS enhances CcpA binding to the promoter region of genes involved in sugar uptake, alternative metabolism, sporulation, and toxin production^18^.

A global mapping of CcpA targets in *C. difficile*, cultured in tryptone–yeast extract (TY) with or without 0.5% glucose, demonstrates that CcpA directly controls ~140 genes by recognizing an “RRGAAAANGTTTTCWW” motif^18^. This analysis reveals that repression of toxin production by CcpA is through direct interactions with the promoter regions of *tcdR*, *tcdA*, and *tcdB*. Glucose repression of toxin production is mediated by CcpA, such that deletion of *ccpA* in *C. difficile* causes constitutive toxin production in the presence of glucose. However, more regulatory factors seem to be involved in the CcpA regulation of toxin production, as the toxin titer in the *ccpA* mutant is lower than in the wild‐type with glucose. Besides toxin production, CcpA also reduces the expression of *spo0A*, which encodes the master regulator of sporulation, via direct binding to its promoter region^18^. Unlike toxin production, glucose suppresses sporulation independent of CcpA, and the regulation of sporulation by CcpA and glucose has no synergistic effects.

### CodY

CodY is a highly conserved global transcriptional regulator in low G+C Gram‐positive bacteria, repressing genes involved in various alternative metabolic pathways during the exponential phase, and the repression is relieved in the stationary phase^19^. CodY is a dimeric protein that consists of an N‐terminal effector binding domain and a winged C‐terminal DNA binding helix‐turn‐helix domain^19^. Upon binding to its effectors, branched‐chain amino acids (BCAAs) and GTP, the affinity of CodY to its targets increases, leading to the repression of genes involved in alternative metabolisms^19^. When BCAA and GTP levels decrease, which serves as an indication of the poor nutritional state, CodY no longer binds to these effectors and de‐represses its regulon.

More than 100 genes are regulated directly or indirectly by CodY^20^. Specifically, CodY binds to the promoter region of *tcdR* and represses toxin production in the presence of GTP or BCAAs^21^. Yet, only weak interactions have been observed between CodY and the promoter regions of *tcdA* and *tcdB*, suggesting the CodY‐mediated toxin regulation is primarily through the toxin‐specific sigma factor TcdR^21^. CodY is also a negative regulator of sporulation, as deletion of *codY* increases the sporulation frequency in *C. difficile*, although the fold change is strain‐specific^22^. Besides, the expression of sporulation‐specific genes in *codY* mutants is increased. The mechanism of CodY‐mediated sporulation regulation remains unclear since the *spo0A* gene is not directly regulated by CodY^20^. CodY may regulate sporulation by modulating the expression of the *opp* operon, which encodes oligopeptide transporters, and/or *sinR*, which encodes a regulator of sporulation described below^22^.

### SinR and SinR′

The *sinRI* locus in *Bacillus subtilis* is comprised of SinR, a master regulator of biofilm formation and inhibitor to sporulation, and SinI, the SinR antagonist^23^. SinR in *B. subtilis* is a tetrameric repressor composed of an N‐terminal helix‐turn‐helix DNA binding domain and a C‐terminal multimerization domain^23^. However, the *sinRR′* locus in *C. difficile* contains two BsSinR homologs, SinR and SinR′, without SinI, suggesting an analogous physiological role^24^, ^25^. RNA sequencing shows SinRR′ in *C. difficile* controls a wide range of genes involved in sporulation, toxin synthesis, motility, and various metabolic pathways. Specifically, overexpression of *sinR* promotes sporulation and toxin production, while SinR′ interacts with SinR and inhibits its function^24^, ^25^. The regulatory effect of SinR on sporulation or toxin production may be through CodY, as SinR binds to the upstream region of *codY* and CodY expression increases 3–30‐fold in *sinRR*′ mutants^24^, ^25^. Interestingly, CodY also binds to the *sinRR*′ promoter region and inhibits its transcription, forming a toggle switch in *C. difficile* ^20^, ^24^, ^25^. When entering the stationary phase, CodY‐mediated repression of *sinRR*′ is alleviated as BCAAs and GTP become less abundant. This leads to increased production of SinR, which can further repress the expression of *codY* and thus alter sporulation initiation and toxin production. In addition, *sin* locus expression is repressed by Spo0A, which directly binds to the promoter region of the locus^26^.

## REGULATORS CONTROL TOXIN PRODUCTION AND SPORULATION IN OPPOSITE DIRECTIONS

### SigH

Numerous bacteria have more than one sigma factor to adapt to the environmental and cellular nutritional state changes. SigH is a conserved factor in many *Firmicutes* that is produced during the transition from exponential to stationary phase, which governs gene expression related to motility, sporulation, virulence, and so forth^27^. SigH positively regulates *spo0A* and genes involved in sporulation by recognizing a (A/G)NAGGA(A/T)3‐N11‐12‐(A/G)NNGAAT motif. Accordingly, the inactivation of SigH completely abolishes sporulation in *C. difficile* ^27^. Additionally, SigH and Spo0A reciprocally activate each other, creating positive feedback to reinforce the sporulation^27^, ^28^. Conversely, the *C. difficile* PaLoc is negatively regulated by SigH, such that the expression of *tcdR*, *tcdA*, *tcdB*, and *tcdE* are increased in a *sigH* mutant. The SigH recognition sequence is not identified in the promoters of these genes, indicating that the regulation is likely indirect^27^.

### Spo0A

Spo0A is a highly conserved master regulator of sporulation in both the *Bacilli* and *Clostridia* ^29^, ^30^. In *B. subtilis*, Spo0A is phosphorylated by sensor histidine kinases (KinA, KinB, and KinC) via phosphorelay^30^. Upon phosphorylation, Spo0A is activated and initiates the sporulation process^30^. In *C. difficile*, the phosphorelay is missing, but Spo0A is phosphorylated by a yet‐to‐be‐identified organ kinase, which then starts the sporulation process similar to *B. subtilis* ^31^. Unlike sporulation, the role of Spo0A in toxin production is controversial. One report demonstrates inactivation of *spo0A* results in a significant decrease in toxin production in *C. difficile* 630*Δerm* (RT012/ST54), an erythromycin‐sensitive derivative of the reference strain 630^32^, ^33^. On the contrary, another study shows that the effects of Spo0A on toxin production are strain‐specific, such that Spo0A is a negative regulator of toxins in certain *C. difficile* strains such as R20291 (RT027/ST1), M7404 (RT027), and JGS6133 (RT078), but not in 630*Δerm* ^34^. This could be due to the genome variation among different strains, as the *C. difficile* with a core gene set only ~20%^34^.

### TcdR

TcdR is a σ^70^ family RNA polymerase sigma factor that recruits RNA polymerase to activate toxin genes and itself^35^. Therefore, the deletion of *tcdR* severely downregulates toxin production by decreasing the expression of *tcdA*, *tcdB*, *tcdR*, and *tcdE* ^36^. However, the effect of TcdR on sporulation is also strain‐specific. The sporulation efficiency in the *tcdR* mutant of *C. difficile* 630 is almost a twofold increase over the parental strain^36^. On the contrary, in *C. difficile* R20291, a nearly threefold reduction of sporulation efficiency is observed in the *tcdR* mutant compared to its parental strain^36^. Consistently, the expression of sporulation‐related genes is downregulated in the absence of TcdR in R20291. Interestingly, it has been demonstrated that a subset of *C. difficile* cells is able to sporulate and produce TcdA^37^, ^38^. The possibility cannot be ruled out that toxin and sporulation gene expression are sequential events, such that toxin production is interrupted by sporulation, and, therefore, a subset of cells appears to both induce toxin gene expression and sporulate^37^. In addition, *C. difficile* growth on a solid surface of TY plates or SMC plates seems to reduce the population of both toxin production and sporulation^38^. This may suggest that quorum sensing plays a role in creating a division of labor between toxin production and sporulation since autoinducers are almost evenly distributed in the growth medium with constant shaking, whereas these molecules may have gradients inside colonies growing on the plate.

### RstA

RstA is a multifunctional transcriptional regulator that contains a conserved N‐terminal helix‐turn‐helix DNA‐binding domain, five TPR repeat regions, and a C‐terminal putative quorum sensing domain^39^. In *C. difficile* 630*Δerm*, the expression of *rstA* is auto‐regulated by recognizing a 29‐bp imperfect repeat within its −10‐consensus sequence^39^, ^40^. RtsA positively regulates spore formation, such that the *rstA* mutant expresses sporulation‐related genes at reduced levels compared to the WT strain^38^, ^39^. The specific regulatory mechanism is unknown, but the TPR and C‐terminal quorum sensing domain likely plays a role since complementation of RstAΔHTH in *rstA* mutant can partially restore *C. difficile* sporulation^39^. On the contrary, RstA negatively regulates toxin production. Studies in *C. difficile* 630*Δerm* show that RstA directly binds to the promoter regions of *tcdA*, *tcdB, tcdR*, and *sigD* and represses the expression of these genes^38^, ^39^, ^40^. Additionally, the regulation by RstA is strain‐specific, as the regulatory effects of RstA on sporulation and toxin production are similar but more robust in *C. difficile* R20291 than 630*Δerm* strain^41^. This may be caused by stronger autoregulation of RstA in R20291^41^.

### Agr system

The prototypical Gram‐positive bacterial accessory gene regulator (Agr) system in *Staphylococcus aureus* contains AgrB, AgrD, AgrC, and AgrA^42^. The transmembrane protease AgrB processes the AgrD peptide to AIP, which is secreted to the extracellular space and bound to AgrC, a membrane‐associated sensor histidine kinase^42^. Upon binding to AIP, AgrC is auto‐phosphorylated and activated, which subsequently phosphorylates the response transcriptional regulator, AgrA. Interestingly, three different Agr systems have been identified in varied *C. difficile* strains^43^: Agr1 only contains AgrB1 and AgrD1, which are found in all sequenced *C. difficile* strains; Agr2, found in R20291, contains the entire prototypical ABCD genes; and Agr3, found in *C. difficile* NAP07 (RT078/ST11) and NAP08 (RT078/ST11) strains, contains AgrC3, AgrB3, and AgrD3, but lacks the AgrA3 response regulator. Deletion of AgrB1, AgrD1, or both in *C. difficile* 630 strain causes a significant reduction in the sporulation rate, but supernatant from wild‐type cultures restores sporulation. The regulatory effects of Agr1 on toxin production remain elusive. One report demonstrates that the *argB1D1* mutant is not able to produce TcdA and TcdB and is consistently avirulent in mice^44^. However, another report shows that the deletion of *argB1* leads to a significant increase in *tcdR* and *tcdA* transcript levels^43^.

### SigD

SigD regulates ~100 genes, including genes involved in motility, metabolism, and regulation^45^. Specifically, SigD positively regulates *tcdA*, *tcdB*, and *tcdR* via direct binding to the promoter region of *tcdR* ^45^. The presence of high levels of C‐di‐GMP reduces toxin production through SigD^46^. The regulatory effects of SigD on sporulation remain unclear. However, the expression of *sinR* is increased by ~4‐fold in a *sigD* mutant, suggesting a negative regulation on sporulation^45^.

## INTERNAL CONNECTIONS BETWEEN TOXIN PRODUCTION AND SPORULATION IN *C. DIFFICILE*

*C. difficile* infection represents a great health burden in recent years, especially recurrent infections^4^. While toxins are the major pathogenic factors, spores are important causes of persistence and possibly recurrence, and plenty of research suggests a link exists between these two processes^10^, ^11^, ^19^.

Two important questions are yet to be answered. First, are these two cellular processes mutually exclusive? Both toxin production and sporulation require a large amount of energy investment. Therefore, it is intuitively contradictory for a sporulating *C. difficile* cell to produce toxins. However, there are two types of relationships between toxin production and sporulation. In the type I relationship, toxin production is inversely related to sporulation. For example, the sporulation and toxin production in *Bacillus anthracis* are inversely linked via AtxA^47^. Another example is *B. thuringiensis*, which follows the division of labor pattern^48^. In the type II relationship, toxin production is associated with sporulation^49^. For instance, the production of *Clostridium perfringens* enterotoxin (CPE) in the mother cell is coupled with spore formation, via SigE and SigK‐dependent promoters^49^. Consistently, CPE release relies on the lysis of the mother cell at the final stage of sporulation. In *C. difficile*, most of the cells choose to either sporulate or produce toxin, following a division of labor mode^37^, ^38^. However, a small population is able to express TcdA while sporulating^37^, ^38^. Toxins of this small population may be released with spores when the mother cell is lysed. Alternatively, a possible explanation is that toxin and sporulation gene expression are sequential events; therefore, it is difficult to exclude the residue gene expression of the previous cellular process. Further experiments at the single‐cell level may answer this question and reveal the precise mechanisms.

The second question is, what are the environmental cues for toxin production and sporulation? The regulatory effects of key factors on toxin production and sporulation are summarized in Table 1 and Figure 1. It is not surprising that transcriptional regulators involved in carbohydrate and amino acids metabolism tune the two cellular processes in the same direction at the onset of the stationary phase^12^, ^17^, ^18^, ^20^, ^21^, ^22^. However, the environmental cues to differentially regulate these two processes are yet to be revealed. Quorum sensing may be one of the targets. First, the quorum sensing‐related Agr1 system and RstA regulate these two processes differentially^38^, ^39^, ^40^, ^41^, ^42^, ^43^. Second, the division of labor is more obvious in *C. difficile* on a solid surface than in a liquid culture^38^. The autoinducer in the liquid medium is evenly distributed and may not be able to form a gradient as the colony or biofilm does, which may be required to decisively make the decision. Last but not least, most of the research has been done in a laboratory setting. However, the environment in a host is very different from a lab medium. Therefore, toxin production and sporulation mode of *C. difficile* cells need to be tested in a murine infection model or using ex vivo samples. Better study tools and methods may be required to characterize the toxin production and sporulation in an infection‐relevant context.

**Table 1:** Regulatory factors that affect sporulation and toxin production.

		Sporulation		Toxin production		
Factor	Function in *Clostridioides difficile*	Effect	Mechanism	Effect	Mechanism	References
CcpA	The master regulator of carbon catabolite repression (CCR)	Negative	Directly binding to the promoter regions of *tcdR*, *tcdA*, and *tcdB*	Negative	Directly binding to the promoter region of *spo0A*	[17, 18]
CodY	A global transcriptional regulator regulating genes involved in various alternative metabolic pathways during the exponential phase	Negative	Directly binding to the promoter region of *tcdR*	Negative	Unclear, possibly via the *opp* oligo‐peptide transporter operon and the *sinR* regulatory gene	[19, 20, 21, 22]
SinRR′	SinR controls a wide range of genes involved in sporulation, toxin synthesis, motility, and various metabolic pathways; SinR′ is an antagonist to SinR	Positive	Possibly through CodY, as SinR binds to the upstream region of *codY*	Positive	Possibly through CodY, as SinR binds to the upstream region of *codY*	[23, 24, 25]
SigH	An alternative sigma factor during the transition from exponential to stationary phase	Positive	Directly binding to the promoter regions of *spo0A* and sporulation genes	Negative	Unclear, possibly through indirect regulation	[27]
Spo0A	The master regulator of sporulation	Positive	Phosphorylation of Spo0A activates sporulation	Conflicting	Unclear	[26, 29, 31, 32]
TcdR	An alternative sigma factor for toxin production	Conflicting	Unclear	Positive	Directing RNA polymerase to the promoter regions of *tcdA* and *tcdB*	[35, 36]
RstA	Affecting sporulation initiation, toxin production, and motility	Negative	Directly binding to the promoter regions of *tcdR*, *tcdA*, *tcdB*, and *sigD*	Positive	Unclear, possibly through the TPR and C‐terminal quorum sensing domain	[37, 38, 39, 40, 41]
Agr system	Quorum sensing system, involved in toxin expression, motility, and sporulation	Positive	Unclear	Conflicting	Unclear	[42, 43, 44]
SigD	An alternative sigma factor, regulates genes involved in motility, metabolism and regulation	Positive	Directly binding to the promoter region of *tcdR*	Unclear	Unclear	[45, 46]
